# The association between hyperandrogenemia and the metabolic syndrome in morbidly obese women

**DOI:** 10.1186/s13098-015-0040-5

**Published:** 2015-05-21

**Authors:** T.G. Valderhaug, J.K. Hertel, N. Nordstrand, P.O. Dale, D. Hofsø, J. Hjelmesæth

**Affiliations:** Morbid Obesity Centre, Vestfold Hospital Trust, Tønsberg, Norway; Division of Medicine, Department of Endocrinology, Akershus University Hospital HF, Sykehusveien 25, 1478 Nordbyhagen, Norway; Division of Medicine and Laboratory Sciences, Institute of Clinical Medicine, University of Oslo, Oslo, Norway; Department of Surgery, Vestfold Hospital Trust, Tønsberg, Norway; Department of Endocrinology, Morbid Obesity and Preventive Medicine, Institute of Clinical Medicine, University of Oslo, Oslo, Norway

**Keywords:** Morbid obesity, Insulin resistance, Hyperandrogenemia, Metabolic syndrome

## Abstract

**Background:**

Female abdominal obesity is associated with hyperandrogenemia (HA), but few studies have addressed the possible association between HA and metabolic syndrome (MetS) among obese women. Some studies indicate that insulin resistance may cause HA through different mechanisms. On the other hand, a bidirectional relationship between HA and insulin resistance has been suggested. Thus, we aimed to investigate if morbidly obese women with HA had higher odds of MetS and its components than those without HA (controls), independent of polycystic ovarian syndrome (PCOS) status.

**Methods:**

This cross-sectional study comprised 1900 consecutive treatment seeking morbidly obese women <50 years. Free testosterone index (FTI) >0.6 defined HA. Women with previously diagnosed PCOS and those with oligo- / anovulation combined with clinical or biochemical hyperandrogenism were defined as having PCOS. Multiadjusted associations between HA and MetS were assessed by logistic regression analysis.

**Results:**

Out of 1900 morbidly obese women, 1089 (57 %), 846 (45 %) and 312 (16 %) had MetS, HA and PCOS, respectively. Compared with controls (without HA), women with HA were younger (34 [1] years vs. 39 [2], *p* < 0.001) had a higher prevalence of MetS (62 % vs. 53 %, *p* < 0.001), type 2 diabetes (18 % vs. 15 %, *p* = 0.045), low HDL-cholesterol (65 % vs. 48 %, *p* < 0.001) and hypertriglyceridemia (48 % vs. 41 %, *p* = 0.004), but a lower prevalence of raised blood pressure (53 % vs. 59 %, *p* = 0.014). Multivariable analyses showed that HA was associated with increased odds of MetS (OR 1.61 [95 % CI 1.27, 2.02]), dysglycemia (1.65 [1.28, 2.11]), low HDL-cholesterol (1.58 [1.27, 1.97]), and hypertriglyceridemia (1.43 [1.15, 1.79]). After stratification for the presence of PCOS, the results remained largely unchanged in women without PCOS; MetS (1.52 [1.18, 1.96), dysglycemia (1.71 [1.30, 2.25]), low HDL-cholesterol (1.55 [1.22, 1.98]) and hypertriglyceridemia (1.36 [1.06, 1.74]).

**Conclusion:**

Morbidly obese women with HA had an approximately 1.5-fold increased odds of having MetS even in the absence of PCOS. Randomized controlled clinical trials, including therapeutic strategies to lower free testosterone levels, are however necessary to explore any cause-and-effect relationship.

## Background

The polycystic ovarian syndrome (PCOS) is the most common female endocrinopathy, affecting 8 – 12 % of women of reproductive age [1], and 10–35 % of obese women [2–4]. PCOS is associated with infertility, hyperandrogenemia, impaired glucose tolerance and type 2 diabetes [5]. A combination of increased levels of androgens and insulin is believed to contribute to the pathophysiology of PCOS [5]. Hyperandrogenemia (HA) comprises the biochemical hallmark of PCOS with elevated free testosterone levels accounting for the majority of the abnormal laboratory findings in women with oligomenorrhea [6].

Excess body weight is associated with HA [7]. Furthermore, the hyperinsulinemia in obese women may directly increase free testosterone levels by lowering the sex hormone binding globulin synthesis in the liver [8]. On the other hand, rodent models have shown that HA promotes insulin resistance, reduces energy expenditure and, accordingly, increases the risk of abdominal obesity and metabolic risk factors [9, 10]. In a multiethnic sample of more than 2500 U.S. women between 42 and 52 years of age, oligomenorrhea was associated with the metabolic syndrome (MetS) only when coincident with HA [11]. Conversely, women with HA had a significantly increased risk of the MetS independent of the menstrual frequency status [11].

The ovaries’ response to luteinizing hormone (LH) is the main source of increased androgens in PCOS [12]. Also, the elevated response of adrenal steroids in women with PCOS sustained only with adrenal stimulation of ACTH, suggesting a secondary effect of increased adrenal androgen production rather than adrenal congenital enzyme deficit [13].

It is not clear how HA may affect cardiovascular disease. Abdominal obese women with PCOS are considered at high risk of cardiovascular disease, and a positive association between coronary artery disease and clinical hyperandrogenism (hirsutism and acne) has been reported [14]. On the other hand, a population based study showed that premenopausal overweight women with PCOS did not have a higher risk of coronary artery disease than those without PCOS [15]. Nevertheless, women with PCOS and HA have a higher prevalence of obesity and adverse metabolic abnormalities compared to those without HA [16]. To the best of our knowledge, no previous study has assessed the impact of HA on the MetS independent of the presence or absence of PCOS among obese women.

The primary aim of this study was to examine whether treatment seeking premenopausal morbidly obese women with HA had higher odds for MetS and its components (low HDL-cholesterol, hypertriglyceridemia, raised blood pressure, and dysglycemia) than women without HA, independent of PCOS status.

## Methods

### Design and study population

A total of 2681 consecutive treatment seeking morbidly obese women attending the Morbid Obesity Centre at Vestfold Hospital Trust, Norway, during the period from November 28^th^ 2005 until July 28^th^ 2014 were assessed for eligibility. To avoid biological bias of including both pre- and postmenopausal women, we excluded 743 women ≥50 years [17–19]. Data on HA were missing for 38 women (PCOS absent n = 34 and PCOS present n = 4), leaving 1900 morbidly obese women of reproductive age to be included in this cross-sectional analysis. The study was approved by the Regional Committee for Medical and Health Research Ethics (S-05175). The participants were included after providing written informed consent, and the study was performed in accordance with the Declaration of Helsinki [20].

### Definitions

We defined MetS according to the joint interim statement of the International Diabetes Federation Task Force on Epidemiology and Prevention; National Heart, Lung, and Blood Institute; American Heart Association; World Heart Federation; International Atherosclerosis Society; and International Association for the Study of Obesity (2009); if WC ≥ 80 cm combined with a minimum of two out of four criteria present: 1) low HDL-cholesterol; HDL-cholesterol <1.3 mmol/L, 2) hypertriglyceridemia; triglycerides ≥1.7 mmol/L, 3) raised blood pressure; systolic blood pressure ≥130 mmHg or diastolic blood pressure ≥85 mmHg, or use of blood pressure lowering medication, and 4) dysglycemia; fasting serum glucose ≥5.6 mmol/L or diabetes mellitus [21]. Type 2 diabetes was diagnosed in patients who had a prior history of type 2 diabetes or a fasting plasma glucose ≥7.0 mmol/L or an HbA1c ≥6.5 %. The Homeostasis Model Assessment Insulin Resistance (HOMA IR) was calculated as ([fasting serum glucose (mmol/l) * fasting serum insulin (pmol/l)]/135) [22]. We calculated the free testosterone index (FTI) using the formula: FTI = 100 x serum testosterone (nmol/L) / sex hormone binding globulin (SHBG, nmol/L). Free testosterone was calculated from the formula described by Vermeulen et al. [23]. According to the Androgen Excess & PCOS Society consensus statement on PCOS diagnosis, HA is defined by increased levels of free testosterone [6, 24]. Accordingly, women with an FTI above the upper normal range (FTI > 0.6) were defined as having HA [25]. Women with previously diagnosed PCOS and those with an FTI > 0.6 or hirsutism combined with oligo- / anovulation were defined as having PCOS [16, 26].

### Data collection

Patients had their weight and height measured wearing light clothing, without shoes, and BMI was subsequently calculated (kg/m^2^). We measured WC midway between the lowest rib margin and the iliac crest. Blood pressure was measured with an appropriate cuff after at least 5 minutes rest with the patient seated in an upright position. Three measurements were registered and the average of the second and the third measurement was used in the study. All anthropometric and blood pressure measurements were performed by trained study personnel.

### Laboratory analysis

Blood samples were obtained by venipuncture after an overnight fast and collected in Vacutainers® gel tubes. Serum was separated from cells within two hours.

Analyses of serum glucose, magnesium, uric acid, and blood lipids were performed using dry reagent slide technology on the Vitros 950 Analyzer until November 2006 and thereafter on the Vitros FS 5.1 (Ortho-Clinical Diagnostics, New York, USA). Intact PTH was measured using an electro-chemiluminescence immunoassay on the Elecsys 2010 (Roche Diagnostics GmbH) or Architect i1000SR (Abbott Diagnostics). The results of the electro-chemiluminescence immunoassay were multiplied by a conversion factor of 1.34 [27]. Glycosylated hemoglobin was analyzed by high performance liquid chromatography (HPLC) using Tosoh HLC-723 G7 (Tosoh Corporation, Tokyo, Japan). All other analyses were performed on the day of blood sampling at the Department of Clinical Chemistry at Vestfold Hospital Trust. Sera for analysis of insulin, testosterone, SHBG and 25(OH) vitamin D (25-hydroxyvitamin D_2_ + 25-hydroxyvitamin D_3_) were stored at −20 °C and analyzed within one week of blood sampling at the Hormone Laboratory, Oslo University Hospital, Aker. Insulin and 25(OH) vitamin D were analyzed in serum by radioimmunoassay (Linco Research Inc, St Charles, MO, and DiaSorin, Stillwater, MN). The interassay coefficients of variation (CV) for insulin and 25 (OH) vitamin D were 8 % and 14 %, respectively. Testosterone was measured in serum by a competitive radioimmunoassay (RIA) technique using ^125^I testosterone (Orion Diagnostica, Espoo, Finland) with a coefficient of variance (CV) at the lower normal reference interval (mean 2.2 nmol/L) of 11 %. Samples with a testosterone concentration level ≥ 4 nmol/L were checked for interference after ether extraction. SHBG in serum was measured by immunoluminometric assay (ILMA) (Immunlite 2000 or 2500, Siemens, NY, US, Healthcare Diagnostics) with a CV of 6 %.

### Statistical analysis

Data are presented as mean (SD) or proportions. Continuous and categorical variables were compared using independent samples t-test and χ^2^ test or Fisher’s exact test as appropriate. Correlations were calculated with Pearson’s correlations coefficient (r) for normally distributed variables and Spearman’s Rho (ρ) for non-normally distributed variables. Demographic, anthropometric and metabolic variables were analyzed including the whole study population and after stratification for presence or absence of PCOS.

The MetS and its components were modeled as dependent variables and HA as the primary explanatory variable. The association between HA and MetS was adjusted for significant confounders identified using a backward logistic regression approach. Variables with p-values below 0.10 were included in the final model (Wald test). Consequently, coronary artery disease (yes/no), prednisolone usage (yes/no), chronic obstructive pulmonary disease (yes/no), joint pain (yes/no), use of estrogens or gestagen medication (yes/no), and thyroid stimulating hormone were removed from the regression model. The models were adjusted for the following covariates in the final multivariable analysis: age (years), family history of diabetes (yes/no), uric acid (μmol/L), anxiety or depression (yes/no), cholelithiasis (yes/no), history of smoking (current or former/never), physical activity (≥1 hour vigorously physical activity/week), parathyroid hormone (pmol/L), and vitamin D-25-OH (nmol/L). We decided not to adjust for WC and HOMA IR in order to avoid the possibility of over-adjustment bias by variables known to be in the causal pathway between HA and the MetS [10, 28].

Univariate and multivariate logistic regression models were used to assess the associations between HA and MetS and its components (hypertriglyceridemia, low HDL-cholesterol, raised blood pressure, and dysglycemia) in all the 1900 included patients. In addition, the analyses were repeated in a subgroup of obese women without PCOS and another group with PCOS, respectively. Possible effect modifications by age and PCOS were investigated by including the products age*HA, and PCOS*HA as interaction terms in the multivariable analyses with MetS as the dependent variable.

The goodness of fit was tested using the Hosmer and Lemeshow test. P-values below 0.05 were considered statistically significant. However, due to the considerable number of statistical tests performed, particular attention should be directed towards smaller P-values, i.e. those below 0.01. The analyses were implemented using IBM SPSS statistics 20.

## Results

**Fig. 1:**
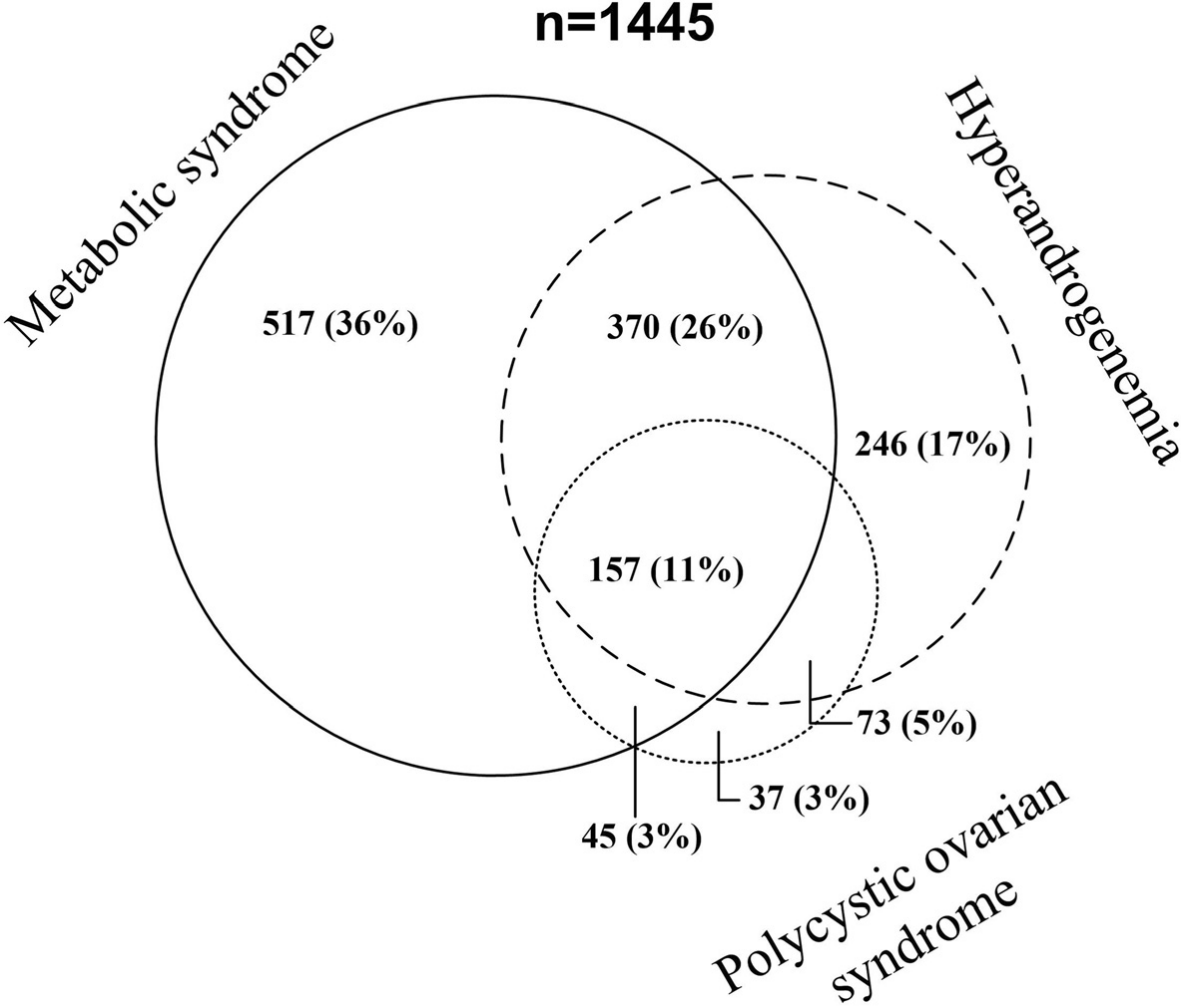
The figure shows the overlap between the 1445 women characterized by their having the metabolic syndrome, polycystic ovarian syndrome and hyperandrogenemia (HA). Women without any of these conditions (n = 455) were not included in Fig. 1

A total of 1089 (57 %), 846 (45 %) and 312 (16 %) out of 1900 morbidly obese women under 50 years of age (mean age 37 [SD 8] years) had MetS, HA and PCOS, respectively. A total of 1445 women had either the metabolic syndrome (MetS), hyperandrogenemia (HA) or polycystic ovarian syndrome (PCOS), whereas 455 women had neither MetS, HA or PCOS. The overlap of women with MetS, HA and PCOS is presented in Fig. 1.

In the whole study population, women with HA were younger (mean [SD] age 34 [1] vs. 39 [2] years, *p* < 0.001), heavier [body weight 125 [19] vs. 121 [18] kg, *p* < 0.001), had a higher WC (127 [13] vs. 123 [13] cm, *p* < 0.001), and were more insulin resistant than the women without HA (HOMA IR; 5.8 [5.5] vs. 4.5 [4.3], *p* < 0.001). The HA group had a significantly higher prevalence of MetS (62 % vs. 53 %, *p* < 0.001), PCOS (27 % vs. 8 %, *p* < 0.001) and were more likely to have low HDL-cholesterol (65 % vs. 48 %, *p* < 0.001), hypertriglyceridemia (48 % vs. 41 %, *p* = 0.004) and type 2 diabetes (18 % vs. 15 %, *p* = 0.045). Women with HA had a lower prevalence of raised blood pressure (53 % vs. 59 %, *p* = 0.014) and were less likely to use blood pressure lowering medication (18 % vs. 25 %, *p* < 0.001). A tendency towards a higher prevalence of dysglycemia was seen in women with HA compared to women without HA (32 % vs. 28 %, *p* = 0.069).

The demographic, anthropometric and metabolic differences between participants with and without HA did not change substantially after stratification according to presence or absence of PCOS (Table 1). The prevalence of MetS was higher in women with HA than in women without HA independent of the presence or absence of PCOS (PCOS absent: 60 % vs. 53 %, *p* = 0.007 and PCOS present: 68 % vs. 55 %, *p* = 0.032).

**Table 1:** Patient characteristic according to presence or absence of polycystic ovarian syndrome (PCOS) and hyperandrogenemia (HA)

	PCOS absent			PCOS present		
	HA present	HA absent	*P* value	HA present	HA absent	*P* value
N	616	972	-	230	82	-
Age, yrs	35 (8)	39 (7)	<0.001	33 (8)	36 (8)	0.001
BMI, kg/m^2^	44.4 (5.8)	43.2 (5.9)	<0.001	44.8 (6.4)	42.8 (6.2)	0.015
Weight, kg	124 (18)	121 (18)	<0.001	126 (20)	119 (19)	0.016
Waist circumference, cm	127 (13)	123 (13)	<0.001	127 (14)	123 (13)	0.025
WHR	0.94 (0.09)	0.92 (0.08)	<0.001	0.94 (0.08)	0.93 (0.10)	0.384
Metabolic syndrome	370 (60 %)	517 (53 %)	0.008	157 (68 %)	45 (55 %)	0.032
-waist ≥ 80 cm	611 (100 %)	963 (100 %)	1.000	228 (100 %)	82 (100 %)	-
-lipids	457 (74 %)	633 (65 %)	<0.001	186 (81 %)	51 (62 %)	0.001
triglycerides ≥1.7 mmol/L	285 (46 %)	398 (41 %)	0.037	116 (50 %)	32 (39 %)	0.094
HDL-cholesterol <1.30 mmol/L	391 (64 %)	469 (49 %)	<0.001	160 (70 %)	41 (50 %)	0.002
lipid lowering medication	37 (6 %)	66 (7 %)	0.601	18 (8 %)	8 (10 %)	0.642
-raised blood pressure	320 (52 %)	570 (59 %)	0.007	131 (57 %)	50 (61 %)	0.603
Systolic blood pressure ≥130, mmHg	233 (38 %)	386 (40 %)	0.428	106 (47 %)	36 (44 %)	0.700
Diastolic blood pressure ≥ 85, mmHg	195 (32 %)	311 (32 %)	0.868	74 (33 %)	28 (34 %)	0.785
Blood pressure lowering medication	110 (18 %)	246 (25 %)	0.001	44 (19 %)	17 (21 %)	0.748
-fasting plasma glucose ≥ 5.6 mmol/L OR diabetes mellitus	192 (31 %)	270 (28 %)	0.157	77 (34 %)	23 (28 %)	0.410
Type 2 diabetes	112 (18 %)	143 (15 %)	0.069	42 (18 %)	12 (15 %)	0.501
Cholelithiasis	59 (10 %)	114 (12 %)	0.187	18 (8 %)	7 (9 %)	0.816
Family history diabetes	195 (32 %)	300 (31 %)	0.781	87 (38 %)	30 (37 %)	0.895
Systolic blood pressure, mmHg	125 (15)	126 (14)	0.290	127 (14)	128 (16)	0.806
Diastolic blood pressure, mmHg	80 (10)	80 (10)	0.394	80 (10)	80 (10)	0.516
Uric acid, μmol/L	345 (69)	325 (69)	<0.001	358 (69)	325 (72)	<0.001
PTH, pmol/L	6.9 (2.9)	7.2 (3.3)	0.045	6.7 (3.0)	7.3 (2.9)	0.147
Vitamin D 25-OH, nmol/L	54 (21)	57 (21)	0.006	49 (19)	50 (18)	0.840
Magnesium	0.84 (0.07)	0.85 (0.07)	0.178	0.83 (0.07)	0.83 (0.07)	0.978
Anxiety or depression	290 (47 %)	446 (46 %)	0.643	122 (53 %)	38 (46 %)	0.306
Insulin, pmol/L	129 (95)	107 (83)	<0.001	142 (75)	118 (70)	0.012
HOMA IR, (mmol/l * pmol/L / 135)	5.7 (6.0)	4.5 (4.4)	<0.001	6.0 (3.9)	4.8 (3.4)	0.017
≥1 hour physical activity/week	329 (64 %)	549 (67 %)	0.238	126 (68 %)	44 (60 %)	0.246
≥1 hour vigorous physical activity/week	192 (37 %)	356 (43 %)	0.026	73 (40 %)	32 (44 %)	0.574
Use of estrogens or gestagen medication	62 (10 %)	112 (12 %)	0.410	15 (7 %)	20 (24 %)	<0.001
Smoke (current or former/never)	368 (60 %)	561 (58 %)	0.432	147 (64 %)	38 (46 %)	0.006
SHBG, nmol/L	22.3 (9.2)	44.3 (29.2)	<0.001	20.8 (8.1)	46.6 (30.3)	<0.001
Total testosterone, nmol/L	1.90 (0.71)	1.16 (0.55)	<0.001	2.3 (1.2)	1.3 (0.5)	<0.001
Estimated free testosterone, nmol/L	0.039 (0.085)	0.003 (0.005)	<0.001	0.056 (0.140)	0.003 (0.005)	0.001
FTI	1.3 (3.1)	0.3 (0.1)	<0.001	1.7 (5.2)	0.3 (0.1)	0.017

BMI; body mass index. WHR; waist hip ratio. PTH; parathyroid hormone. PCOS; polycystic ovarian syndrome. HOMA IR; Homeostasis Model Assessment - Insulin Resistance. FTI; free testosterone index, FTI > 0.6; Hyperandrogenemia (HA). SHBG; sex hormone binding globulin

**Fig. 2:**
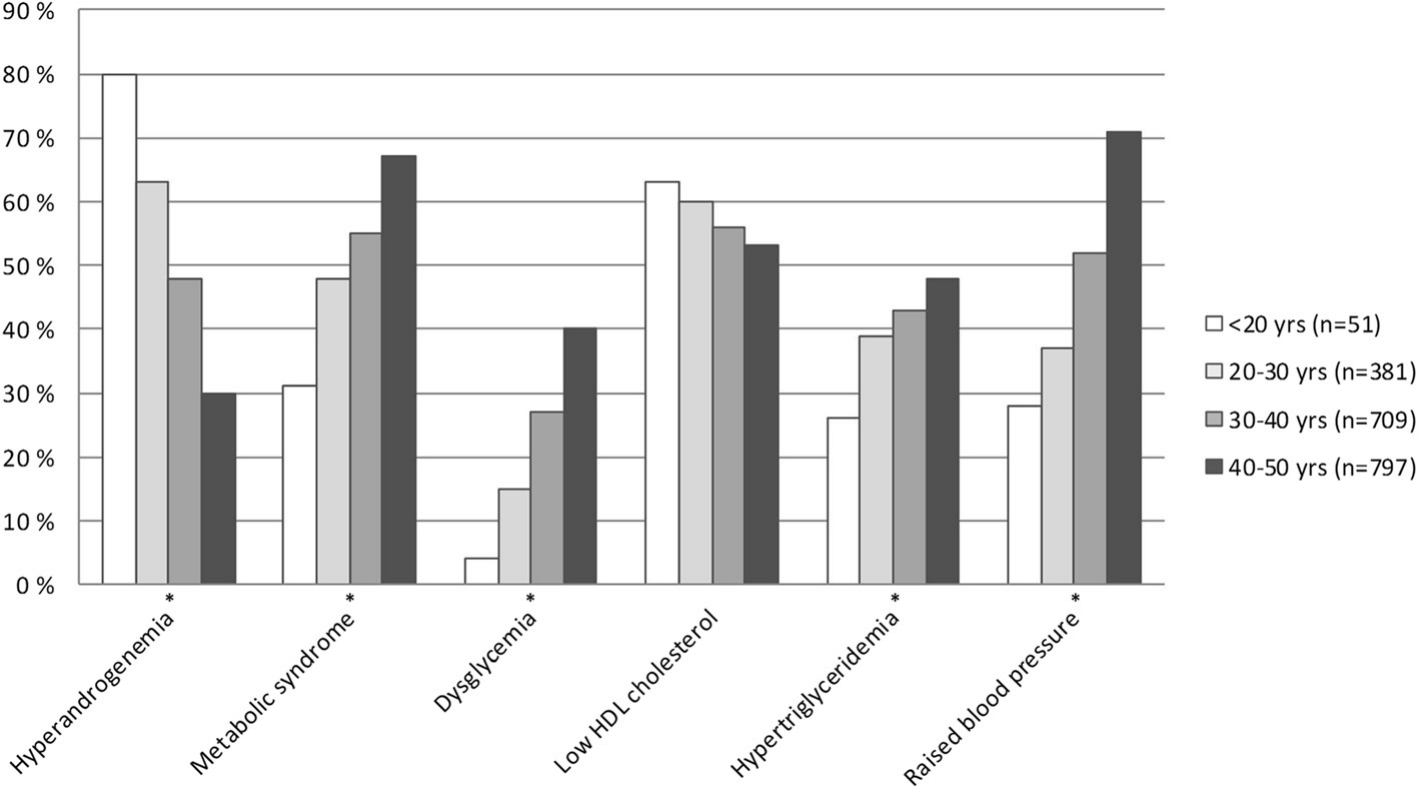
The figure shows the proportion of morbidly obese women with hyperandrogenemia (HA), the metabolic syndrome (MetS) and its components according to various age categories. The prevalence of HA decreased, while the proportions of patients with MetS, dysglycemia, hypertriglyceridemia and raised blood pressure increased significantly with increasing age (* *p* < 0.001). The proportion of women with low HDL-cholesterol did, not change significantly with age (*p* = 0.145)

The proportion of women with HA decreased from 80 % (<20 years) to 30 % (40–50 years) with increasing age deciles (Fig. 2). By contrast, the percentages of women with MetS, dysglycemia, hypertriglyceridemia and raised blood pressure increased significantly with age (all *p* < 0.001) (Fig. 2). The proportion of women with low HDL-cholesterol did, however, not change significantly with age (*p* = 0.145).

The FTI correlated weakly with HDL-cholesterol, systolic blood pressure and diastolic blood pressure (*r* = 0.10, *p* < 0.001; *r* = −0.05, *p* = 0.040; and *r* = −0.06, *p* = 0.007) and HOMA IR (ρ = 0.22, *p* < 0.001), but not with BMI, WC, triglycerides and fasting plasma glucose (*r* = 0.04; *r* = 0.03; *r* = 0.03; *r* = −0.01, all *p* > 0.111). We did not find any significant interactions between HA and age (*p* = 0.834) or between HA and PCOS (*p* = 0.527).

**Fig. 3:**
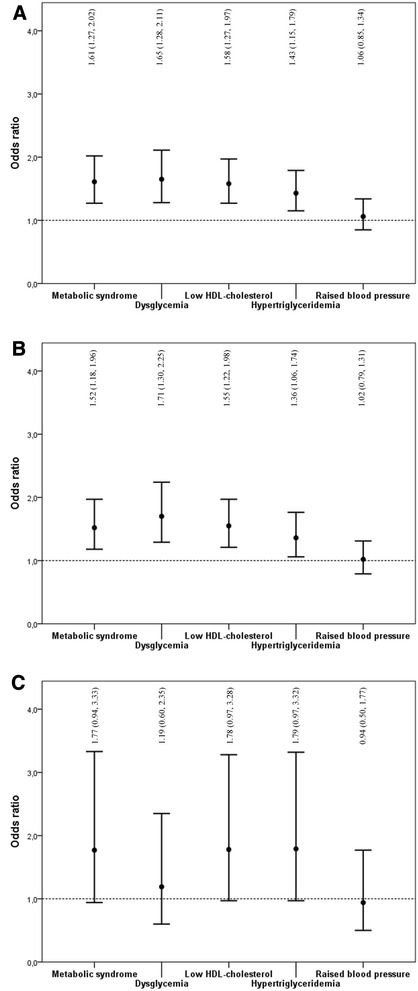
The figure shows the multivariable odds ratios with 95 % confidence intervals (OR [95 % CI]) for metabolic syndrome (MetS) and its components in morbidly obese women with hyperandrogenemia compared to women without hyperandrogenemia (HA) (reference). Panel A comprises all the women included in the study (*n* = 1900), whereas Panel B and C comprises women included in the sub-analyses; PCOS absent (*n* = 1588) and PCOS present (*n* = 312)

In the univariate analysis, HA was associated with a 1.5 fold increased odds of MetS (OR 1.45 [95 % CI 1.20, 1.74]). This association remained statistically significant after adjustments for possible confounders (1.61 [1.27, 2.02]) (Table 2 and Fig. 3A). Moreover, HA was associated with approximately 1.5 fold increased adjusted odds of dysglycemia (1.65 [1.28, 2.11]), low HDL-cholesterol (1.58 [1.27, 1.97]), and hypertriglyceridemia (1.43 [1.15, 1.79]). HA was not associated with raised blood pressure (1.06 [0.85, 1.34]). A sub-analysis of 1588 women without PCOS showed that HA remained significantly associated with MetS (OR 1.52 [95 % CI 1.18, 1.96), dysglycemia (1.71 [1.30, 2.25]), low HDL-cholesterol (1.55 [1.22, 1.98]) and hypertriglyceridemia (1.36 [1.06, 1.74]) (Fig. 3B). In contrast, HA was neither significantly associated with MetS, nor its components in women with PCOS (Fig. 3C).

**Table 2:** The odds of the metabolic syndrome (MetS) and its components adjusted for possible confounders.

	Metabolic syndrome		Dysglycemia		Low HDL-cholesterol		Hypertriglyceridemia		Raised blood pressure	
	OR	95 % CI	OR	95 % CI	OR	95 % CI	OR	95 % CI	OR	95 % CI
Age, yrs	1.06	1.04, 1.07	1.09	1.07, 1.11	1.00	0.98, 1.01	1.03	1.02, 1.05	1.08	1.07, 1.10
Family history diabetes	1.41	1.12, 1.77	1.51	1.19, 1.92	1.24	0.99, 1.54	1.20	0.96, 1.49	1.36	1.09, 1.71
Uric acid, μmol/L	1.01	1.00, 1.01	1.00	1.00, 1.00	1.00	1.00, 1.01	1.01	1.00, 1.01	1.00	1.00, 1.00
Cholelithiasis	1.52	1.05, 2.21	1.25	0.87, 1.80	1.16	0.82, 1.64	1.27	0.90, 1.79	1.24	0.87, 1.78
≥ 1 hour vigorous physical activity/week	0.91	0.73, 1.13	0.95	0.75, 1.20	1.01	0.82,1.25	0.81	0.66, 1.00	0.99	0.80, 1.23
Vitamin D-25-OH, nmol/L	0.99	0.98, 0.99	0.99	0.98, 0.99	0.99	0.98, 0.99	1.00	0.99, 1.00	0.99	0.99, 1.00
PTH, pmol/L	0.98	0.96, 1.01	0.98	0.96, 1.01	0.96	0.94, 0.99	0.97	0.95, 1.00	1.01	0.98, 1.04
Anxiety or depression	1.33	1.08, 1.65	1.43	1.13, 1.80	1.22	0.99, 1.50	1.53	1.24, 1.89	0.91	0.73, 1.12
Smoke (current or former/never)	1.45	1.17, 1.79	1.17	0.92, 1.48	1.38	1.12, 1.70	1.40	1.13, 1.73	0.97	0.78, 1.20
HA, FTI >0.6 (yes/no)	1.61	1.27, 2.02	1.65	1.28, 2.11	1.58	1.27, 1.97	1.43	1.15, 1.79	1.06	0.85, 1.34

Dependent variables; metabolic syndrome and its components. Hypertriglyceridemia; triglycerides; ≥1.7 mmol/L, low HDL-cholesterol; HDL-cholesterol , <1.3 mmol/L, dysglycemia; fasting glucose ≥5.6 mmol/L or established diabetes mellitus, raised blood pressure; systolic blood pressure ≥130 mmHg or diastolic blood pressure ≥85 mmHg or use of blood pressure lowering medication. PTH; Parathyroid hormone. FTI; free testosterone index. HA; hyperandrogenemia. FTI; free testosterone index

## Discussion

The main and novel finding of this study of premenopausal white morbidly obese women, was that HA was associated with 61 % increased adjusted odds of MetS, and that this association was mainly driven by increased odds of dysglycemia and dyslipidemia. Although the prevalence of HA decreased, while the prevalence of MetS increased with increasing age, HA remained an independent predictor of MetS and its components, dysglycemia and dyslipidemia.

### Hyperandrogenemia and the metabolic syndrome

The results of the present study cohere with another cross-sectional study of 2543 multiethnic overweight pre- and perimenopausal women who were on average age ten years older than the participants in the present study (46 vs. 37 years, respectively) [11]. In the previous study, women with HA, regardless of menstrual cycles, had a 1.5 fold increased risk of MetS compared to those without HA [11]. Women with both oligomenorrhea and HA had a 2 fold increased odds of MetS [11]. In our sub-analysis of 1588 severely obese women without PCOS, HA remained a significant predictor of MetS, dysglycemia, low HDL-cholesterol, and hypertriglyceridemia. In contrast, no association between androgen excess, menstrual irregularity and incident MetS or self-reported cardiovascular disease was revealed in a recent study of premenopausal women [28].

### Possible mechanisms linking hyperandrogenemia to insulin resistance and visceral obesity

Animal (rodent) studies indicate that androgens may produce insulin resistance by direct effects on skeletal muscle and adipose tissue, mediated by alterations in the insulin receptor –glycogen synthesis, by altering adipokine secretion, and by increasing visceral adiposity [9]. Moreover, a small study of 13 obese and 30 non-obese women showed that anti-androgen treatment partly reversed the peripheral insulin resistance in non-obese women only, whereas central obesity may have a direct role in androgen hypersecretion [29, 30]. Also, a recent study of young, overweight women suggested that the association between body fat and HA was predominantly mediated by insulin resistance [24]. The interrelationships between body fat, insulin resistance and HA contribute to the complex pattern making it a difficult task to specify the role of each component. Although the findings by Tosi et al. may not be directly comparable with ours, we also report a weak, but significant correlation between insulin resistance and HOMA IR and FTI. Acordingly, although HA was independently associated with MetS, insulin resistance might partly have mediated this effect.

### Association between hyperandrogenemia and dyslipidemia

Our study demonstrated a significant inverse relationship between HA and HDL-cholesterol levels which is in accordance with previous studies of women with PCOS [31, 32]. Reduced HDL-cholesterol levels have also been reported in peri- and postmenopausal women receiving androgen formulations added to hormone replacement therapy and after transdermal testosterone treatment [33, 34]. Furthermore, low levels of SHBG in women with PCOS have been associated with low levels of HDL-cholesterol, independent of insulin resistance and obesity [35]. Conversely, a recent study of young overweight women showed no differences in dyslipidemia in women with HA compared to women without HA [24]. Thus, the impact of HA on dyslipidemia remains unclear and further research is warranted.

### Hyperandrogenemia and dysglycemia

The proportion of women with HA decreased, whereas the proportion of women with dysglycemia increased with age (Fig. 2). Nevertheless, we found a significant association between HA and dysglycemia independent of age.

Studies have shown a high conversion rate from impaired glucose tolerance to diabetes mellitus in women with PCOS [36, 37]. Our findings demonstrated that women with and without PCOS had a comparable prevalence of dysglycemia. However, patients with HA had approximately 65 % increased odds of dysglycemia compared to those without HA, and the association was strengthened after the exclusion of women with PCOS. These findings support the hypothesis that HA may be involved in the pathophysiology of dysglycemia independent of PCOS status.

### Hyperandrogenemia and blood pressure

We did not find any significant association between HA and raised blood pressure. This is in contrast with the results from a study of young normal to overweight Asian women with PCOS which demonstrated that high bioavailable testosterone levels were associated with elevated blood pressure [38]. In another study of middle aged normal- to overweight women, facial hirsutism was associated with higher systolic blood pressure, whereas limb-hirsutism was associated with lower diastolic blood pressure [39]. In the present study, premenopausal morbidly obese women with HA were actually less likely to use blood pressure lowering medication compared to the women without HA (18 % vs. 25 %). Interestingly, a recently published cross-sectional study of normal- to overweight Swedish men and women showed a strong inverse association between blood pressure and SHBG while free testosterone concentration was not associated with hypertension [40]. The authors speculated that SHBG might have a direct effect on the endothelial cells through the receptor for SHBG, but this association was significant only in postmenopausal women ≥50 years of age [40]. In contrast, our study did not demonstrate any association between SHBG and raised blood pressure in premenopausal women (data not shown).

### Strengths and limitations

The major strength of the present study is the large cohort of consecutively included treatment seeking morbidly obese women. The study participants were, however, referred to a tertiary care center for evaluation and treatment with bariatric surgery, medical therapy or long term lifestyle rehabilitation for morbid obesity. Accordingly, the results cannot be generalized to the general obese population (i.e. non-treatment seeking subjects).

We used an immunoassay to measure testosterone. Although conventional immunoassays measure reliable testosterone at high levels, the immunoassays have been reported to be less reliable at low concentrations [41]. However, the separation procedure of diethyl extraction was performed manually and should thus have reduced the number of falsely elevated results.

Furthermore, although we defined FTI >0.6 as HA in the present study, other adrenocortical precursor steroids including pregnenolone, 17-hydroxypregnenolone, dehydroepiandrosterone (DHEA), androstenedione, 11-deoxycortisol and cortisol may have contributed to clinical manifestations of HA. Nevertheless, free testosterone levels remain the predominant laboratory finding in women with oligomenorrhea and the ovaries are the main source of androgen excess in women with and without PCOS [6, 12].

Unfortunately, we did not have precise menopause data. There is some evidence that a higher BMI might cause a later menopause. One study reported that obese women (BMI ≥30 kg/m^2^) had a median age at menopause of 53 years [17]. Consequently, by excluding women over 50 years of age, some morbidly obese premenopausal women might have been left out of our analysis.

Finally, the majority (97 %) of the morbidly obese women were Caucasian, and as such the results of might not be applicable to women of other ethnicities.

## Conclusion

In this study, the prevalences of MetS, PCOS and HA were high among morbidly obese women <50 years of age. Compared to women without HA, those with HA had significantly higher odds of having the MetS, which was mainly explained by the associations between HA and the lipid- and glucose components of the MetS. Our results, if confirmed, suggest that an FTI-blood test might add value to the cardiovascular risk assessment of premenopausal women with morbid obesity. Randomized controlled clinical trials, including therapeutic strategies to lower free testosterone levels, are however necessary to explore any cause-and-effect relationship.

## Abbreviations

BMI: Body mass index
WHR: Waist hip ratio
MetS: Metabolic syndrome
PCOS: Polycystic ovarian syndrome
HOMA IR: Homeostasis model assessment - insulin resistance
FTI: Free testosterone index
HA: Hyperandrogenemia
PTH: Parathyroid hormone
